# Expression characteristics of polymeric immunoglobulin receptor in Bactrian camel (Camelus bactrianus) lungs

**DOI:** 10.1371/journal.pone.0264815

**Published:** 2022-03-04

**Authors:** Wan-hong He, Wang-dong Zhang, Cui-cui Cheng, Jia Lu, Lei Liu, Zhi-hua Chen, Wen-hui Wang

**Affiliations:** College of Veterinary Medicine, Gansu Agricultural University, Lanzhou, Gansu, China; Chang Gung University, TAIWAN

## Abstract

Polymeric immunoglobulin receptor (pIgR), the transmembrane transporter of polymeric immunoglobulin A and M, has multiple immune functions. To explore the characteristics of pIgR expression in Bactrian camel lungs, twelve healthy adult (2–7 years old) Bactrian camels were systematically studied. The results showed that pIgR was mainly expressed in the cytoplasm and membrane of ciliated cells, as well as in the cytoplasm and membrane of basal cells, serous cells of bronchial glands, club cells and alveolar type 2 cells in Bactrian camel lungs. Specially, as the bronchial branches extended, the pIgR expression level in ciliated cells significantly declined (p<0.05), and the corresponding bronchial luminal areas obviously decreased (p<0.05). However, pIgR was not expressed in goblet cells, endocrine cells, alveolar type 1 cells and mucous cells of bronchial glands. The results demonstrated that ciliated cells continuously distributed throughout the whole bronchial tree mucosa were the major expression sites of pIgR, and pIgR was also expressed in basal cells, serous cells of bronchial glands, club cells and alveolar type 2 cells, which would facilitate secretory immunoglobulin A (SIgA) transmembrane transport by pIgR and form an intact protective barrier. Moreover, the pIgR expression level in ciliated cells was positively correlated with the bronchial luminal areas; but negatively correlated with the cleanliness of airflow through the bronchial cross-sections, showing that the pIgR expression level in the bronchial epithelium was inhomogeneous. Our study provided a foundation for further exploring the regulatory functions of immunoglobulins (i.e., SIgA) after transport across the membrane by pIgR in Bactrian camel lungs.

## Introduction

Polymeric immunoglobulin receptor (pIgR), a glycosylated type I transmembrane protein, is mainly composed of an extracellular region, a transmembrane region and an intracellular region. Moreover, the extracellular domain of pIgR contains repeated immunoglobulin-like (Ig-like) domains [1, 2], whose number increases with vertebrate evolution, that is, four in birds, amphibians and reptiles, and five in mammals. Interestingly, pIgR can be expressed in the intestinal tract [3], respiratory tract [4], liver [5], and other organs, but pIgR expression levels are obviously different in the same sites of different animals, in different organs of the same animals, and in different physiological statuses of the same animals. For example, the pIgR expression level in the mouse small intestine is higher after weaning than before [6], while in rats, pIgR expression in small intestine appears only after weaning [7]; the pIgR expression level is significantly higher in the rodent liver than in the respiratory tract [3]; and in diseases such as human lung cancer and rectal cancer, pIgR expression levels are decreased, or even absent [8, 9].

Importantly, pIgR plays a crucial role in mucosal immunity. For example, pIgR can bind and transport polymeric immunoglobulins (pIgs) across the mucosal epithelium by endocytosis [10, 11], then release these pIgs into the luminal mucus layer to form a protective barrier. This is the most important pathway for immunoglobulin transmembrane transport. In humans, pIgA and M can be transported simultaneously by pIgR, but in rodents and birds, only dimer IgA (dIgA, the main type of pIgA) is transported by pIgR [12]. Similar to this pathway, pIg-antigen complexes can be transported into secretions by pIgR [13, 14]. Moreover, secretory component (SC), a proteolytic fragment of pIgR, is also important in mucosal immunity. As the main constitutive structure of secretory immunoglobulin A (SIgA) and M [15, 16], SC can protect these secretory immunoglobulins from proteolytic degradation [17, 18], and SC in free form can neutralize antigens [19, 20]. Moreover, pIgR also has an immunomodulatory function in mediating the intracellular neutralization of dIgA and its antigen [21].

Lung, a place of gas exchange, is continuously exposed to environmental stimuli, however, pIgR expressed on the lung epithelial cells, as the bridge between innate and adaptive immune responses at mucosal surfaces [18] constitutes the first line of lung defense. But unfortunately, few studies have been performed on the pIgR of mammalian respiratory tract, especially in Bactrian camels. More importantly, although there are some reports show that pIgR can be expressed in lungs of human [4, 22–25], pig [26], rat [27, 28], mouse [29], and rhesus monkey [30], which lung cells express pIgR is still under exploration. Therefore, the pIgR expression characteristics in Bactrian camel lungs were systematically analysed by using immunohistochemical, micro-image analysis and statistical methods in this study. We hope that the results of this study will provide support for future exploration of the mucosal immune functions of pIgR in the lower respiratory tract of Bactrian camels.

## Materials and methods

### Ethics statement

All experimental procedures were approved by the Animal Ethical and Welfare Committee of the College of Veterinary Medicine of Gansu Agricultural University (Approval No. GSAU-AEW-2016-0010). The healthy Bactrian camels were provided by the slaughterhouse of Minqin county of Gansu province in China, and the basic diet of camels was provided every day before slaughter, including water (10 L/day) and roughage (16–22 kg/day) [31].

### Experimental animals and group divisions

Twelve healthy adult Alashan Bactrian camels (2 to 7 years old, 6 males and 6 females) were divided into two groups: the lung conducting portion group and the lung respiratory portion group, with 6 camels (3 males and 3 females) in each group. Camels were anesthetized intravenously with sodium pentobarbital (20 mg/kg) and then exsanguinated until death.

### Lung fixation, sampling and microsection

The methods of lung fixation and sampling are described fully in our previous work [32], but briefly, in every camel lung, the sampling locations of the conducting portion were as follows 1. trachea; 2. cranial segmental bronchus of the right anterior lobe (Acr); 3. lobar bronchus of the right anterior lobe; 4. caudal segmental bronchus of the right anterior lobe (Aca); 5. right main bronchus; 6. lobar bronchus of the right posterior lobe; 7. first dorsal segmental bronchus of the right posterior lobe (D1); 8. first lateral segmental bronchus of the right posterior lobe (L1); 9. lobar bronchus of the accessory lobe; 10. ventral segmental bronchus of the accessory lobe (Acv); 11. dorsal segmental bronchus of the accessory lobe (Acd); 12. second dorsal segmental bronchus of the right posterior lobe (D2); 13. second lateral segmental bronchus of the right posterior lobe (L2); 14. fourth dorsal segmental bronchus of the right posterior lobe (D4); 15. third lateral segmental bronchus of the right posterior lobe (L3); 16. fifth dorsal segmental bronchus of the right posterior lobe (D5); 17. sixth dorsal segmental bronchus of the right posterior lobe (D6); 18. cranial segmental bronchus of the left anterior lobe (Acr); 19. lobar bronchus of the left anterior lobe; 20. caudal segmental bronchus of the left anterior lobe (Aca); 21. left main bronchus; 22. lobar bronchus of the left posterior lobe; 23. first dorsal segmental bronchus of the left posterior lobe (D1); 24. first lateral segmental bronchus of the left posterior lobe (L1); 25. second dorsal segmental bronchus of the left posterior lobe (D2); 26. third dorsal segmental bronchus of the left posterior lobe (D3); 27. second lateral segmental bronchus of the left posterior lobe (L2); 28. fourth dorsal segmental bronchus of the left posterior lobe (D4); 29. third lateral segmental bronchus of the left posterior lobe (L3); 30. fifth dorsal segmental bronchus of the left posterior lobe (D5); and 31. sixth dorsal segmental bronchus of the left posterior lobe (D6). In addition, the sampling location of the respiratory portions in each camel lung included 1. four sample blocks in the left cranial lobe; 2. six in the left caudal lobe; 3. four in the right cranial lobe; 4. six in the right caudal lobe and two in the accessory lobe, which were randomly extracted from each lung. The samples were then made into paraffin sections (4 μm), which were stained with haematoxylin and eosin (H&E) and streptavidin biotin complex (SABC) for immunohistochemistry (IHC).

### SABC-IHC staining

#### Primary antibody selection

The structure of pIgR is highly conserved (especially in the transmembrane region); moreover, bioinformatics analysis of pIgR in our laboratory [33] has shown that the primary, secondary and tertiary structures of Bactrian camel pIgR are all highly similar to those of humans. For example, there are five repeated Ig-like domains in the extracellular domain of Bactrian camel pIgR, and the consistency of the amino acid sequence of this domain with that of humans is 69.78%. Hence, the epitopes of pIgR were similar among Bactrian camels and humans. In addition, when the antigenic amino acid sequence of the preparative rabbit polyclonal antibody against human pIgR (Lot No. HPA006154, Sigma, the United States) was compared with the protein sequence of Bactrian camel pIgR in DNAMAN, the similarity of the corresponding regions was 63.89%. According above characteristics, this primary antibody satisfied the requirements of the sequencing experiment.

#### Selection of the optimal working concentration of the primary antibody

Within according to the concentration range (1:50–1:200) required for the application of the rabbit polyclonal antibody against human pIgR, four concentrations including 1:50, 1:100, 1:150 and 1:200 were tested to compare the colour rendering effect, and the optimal working concentration was found to be 1:200.

#### Staining steps

1. The microsections were deparaffinated and washed; 2. 3% H_2_O_2_ was added for approximately 15 min at room temperature to eliminate endogenous peroxidase activity, and the microsections were then washed with distilled water: 2 min × 3 times; 3. 0.1% trypsin was added for approximately 40 min at 37°C to repair the antigen, and the microsections were washed with distilled water: 2 min × 3 times; 4. 5% BSA was added for approximately 40 min at 37°C without washing; 5. the primary antibody (1:200 rabbit polyclonal antibody against human pIgR) was added to the positive group and allowed to stand for approximately 18 hours at 4°C to allow the antigen and antibody to react fully; for the negative group, PBS solution was added instead of the primary antibody and allowed to stand at 4°C for approximately 18 hours; then, both were washed with PBS 5 min × 4 times; 6. secondary antibody (HRP conjugated goat anti-rabbit IgG (from Easy-to-Use Immunohistochemical Kit, Lot No.07H3OCJ, Boster, Wuhan, Hubei, China) was added, and incubated at 37°C for 40 min, followed by washing with PBS 5 min × 4 times; 7. SABC was added and allowed to stand at 37°C for 30 min, followed by washing with PBS 5 min × 4 times; 8. DAB colour rendering was performed at room temperature and away from light, the colour rendering effect was observed under a light microscope, and the excess colour rendering liquid was then washed away; and 9. the microsections were redyed with a small amount of hematoxylin dyeing solution, sealed with neutral balsam, and stored at 50°C.

### Light microscopy observation

The expression characteristics of pIgR in every bronchial branch, including the dorsal, ventral, medial and lateral bronchiole systems, as well as the expression characteristics of pIgR in the respiratory portion of every lung lobe, were carefully observed using an Olympus DP-71 microscopy system, and 30 sections of each location in each sample were photographed.

### Statistical analysis

#### Bronchial luminal area

In the lung conducting portion group, the luminal diameters of the trachea and main bronchus were directly measured with a Vernier calliper. Five diameters were measured in random directions in each lumen (without cartilage ring), and the average values were obtained. The luminal areas were calculated according to the circular area formula. However, the luminal areas of the lobar bronchus, segmental bronchus, sub-segmental bronchus and small bronchus were measured as follows: 10 sections of each sample were selected randomly, and stained with H&E. Sequential photomicrographs were taken, the pictures of the intact bronchial cross-sections were joined together (Adobe Photoshop CC 2017), and the luminal areas were calculated (Image-Pro Plus 6.0). Finally, using IBM SPSS V.23.0 (SPSS Inc., Chicago, USA), the area differences among bronchial branches in different grades, and the area differences in the different segmental bronchus simultaneously separated from the same lobar bronchus were analysed by one-way analysis of variance (ANOVA) followed by Duncan’s multiple range test. A value of p<0.05 was considered to indicate a significant difference.

#### The mean optical density (MOD) of pIgR in the bronchial ciliated epithelial cells

In the lung respiratory portion group, 5 sections of each sample stained with SABC-IHC were randomly selected, and 3 microscopic fields of each section were randomly observed. Then, pIgR (MOD) of the bronchial ciliated cells in each epithelium was calculated (Image-Pro Plus 6.0) as follows: 1. the image was opened, and optical density correction was performed at a blank position in the image; 2. the measurement parameters (the integrated optical density (IOD) was selected as the measurement value) were set and saved; 3. the bronchial epithelium was manually circled with a paint tool as the measurement area; 4. colour (the HIS parameter was selected) was selected and saved, then the date collector (area (sum) and IOD (sum) were selected) was set; 5. the area and IOD values of the selected bronchial epithelial were measured, the measurement values (there were two sets of data) were read, and the one with the largest area and the one with the smallest IOD were selected; and 6. the MOD value was calculated from the IOD/area. Finally, using IBM SPSS V.23.0, the pIgR (MOD) differences among bronchial branches in different grades and the pIgR (MOD) differences in the different segmental bronchi simultaneously separated from the same lobar bronchi, were analysed by ANOVA followed by Duncan’s multiple range test. A value of p<0.05 was considered to indicate a significant difference.

## Results

### Histological characteristics of the different bronchial branches in Bactrian camels

**Fig 1:**
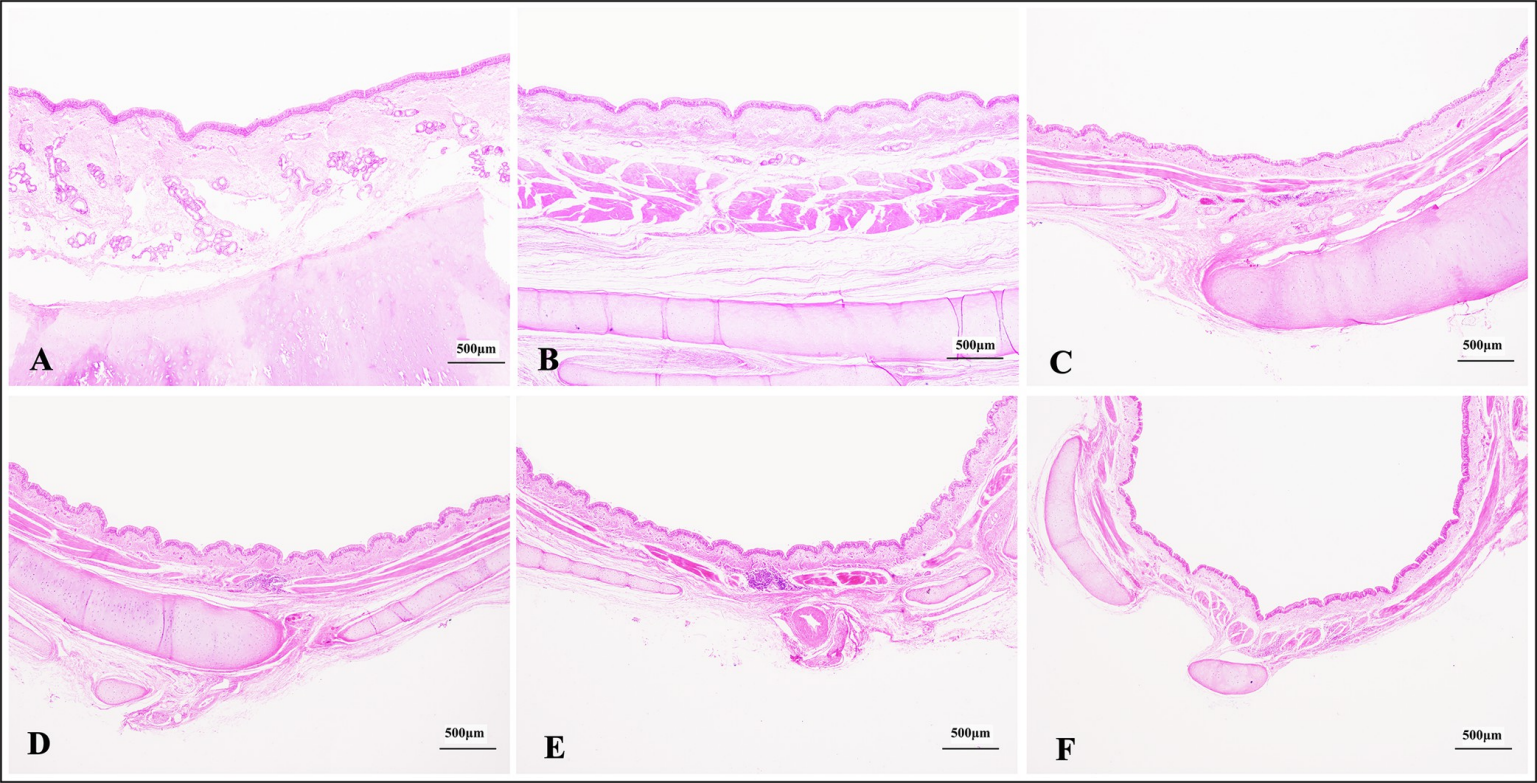
Histological characteristics of the different graded bronchi in Bactrian camels.

As the number of bronchial branches increased, the bronchial wall gradually thinned, the thicker cartilage rings gradually transformed into irregular cartilage pieces, and the number of goblet cells and bronchial glands decreased and nearly disappeared in the bronchioles with simple ciliated columnar epithelium (Fig 1). Specifically, as the bronchial branches extended, the relative luminal areas significantly declined (p<0.05); that is, trachea > main bronchi > lobar bronchi > segmental bronchi > sub-segmental bronchi > small bronchi. In addition, in the different branches at the same grade from the same lobar bronchi, the areas of the larger lumens were significantly higher than those of the smaller lumens (S1 File). For example, Aca > Acr in the left cranial lobe; Aca < Acr in the right cranial lobe; Acv > Acd in the accessory lobe; both in the left and right caudal lobe, L1 > L2 > L3, D1 > D2 > D3 (D3 was absent in right lung) > D4 > D5 > D6, V1 > V2 > V3 > V4 > V5, M3 > M4 > M5 > M6.

In trachea (A) and main bronchi (B), there were thicker bronchial wall, abundant bronchial glands between the epithelial basal lamina and cartilage, and obvious cartilaginous ring; in lobar bronchi (C), segmental bronchi (D), sub-segmental bronchi (E) and small bronchi (F), the bronchial luminal diameter was gradually smaller, the bronchial wall changed thinner, the bronchial glands number became lesser, and the irregular cartilaginous pieces were thinner, smaller and lesser. Pictures (A-F) were all stained with haematoxylin and eosin (H&E), and the original magnifications were all 40×.

### pIgR expression characteristics in the conducting portion of Bactrian camel lungs

**Fig 2:**
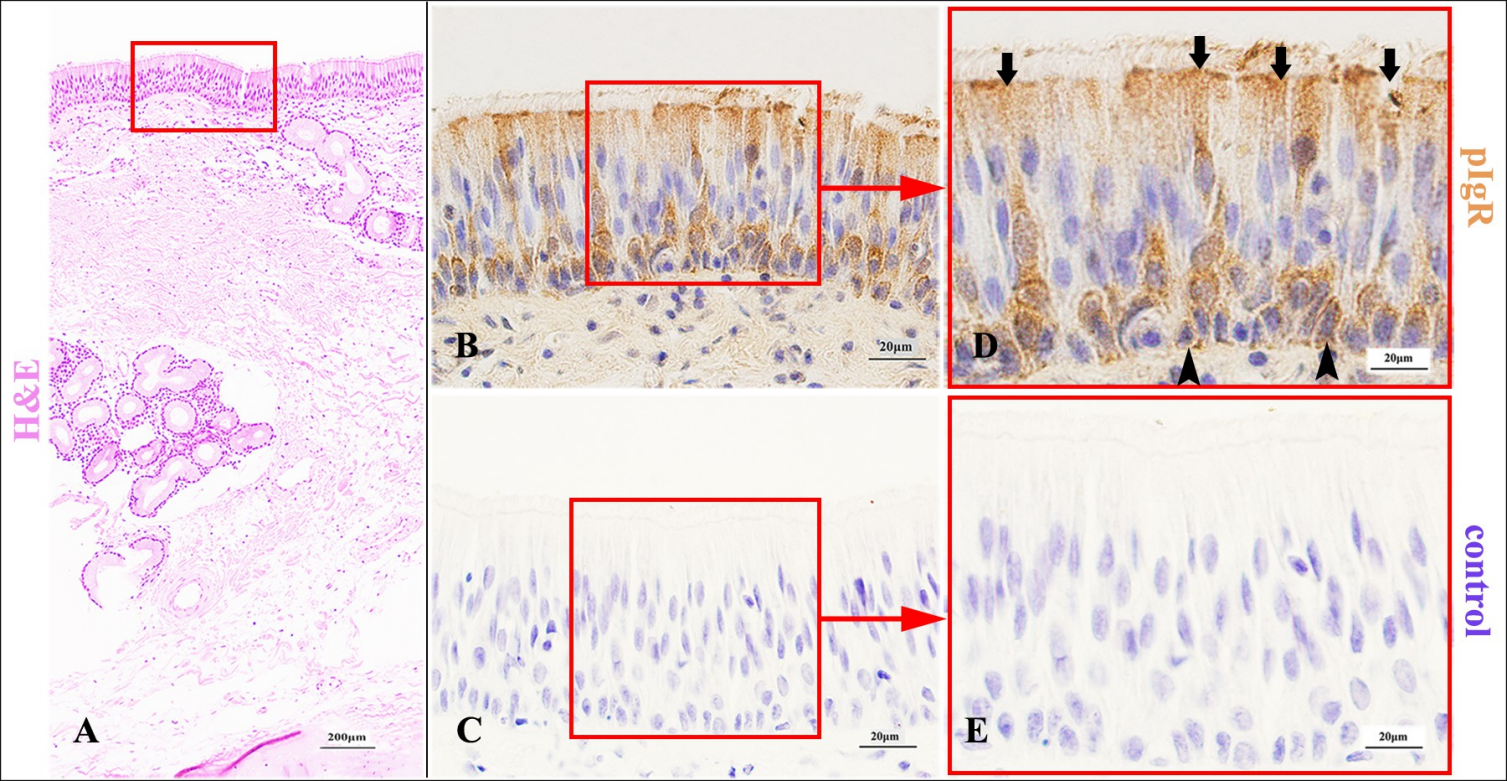
pIgR expression characteristics in the main bronchial epithelium of Bactrian camels.

**Fig 3:**
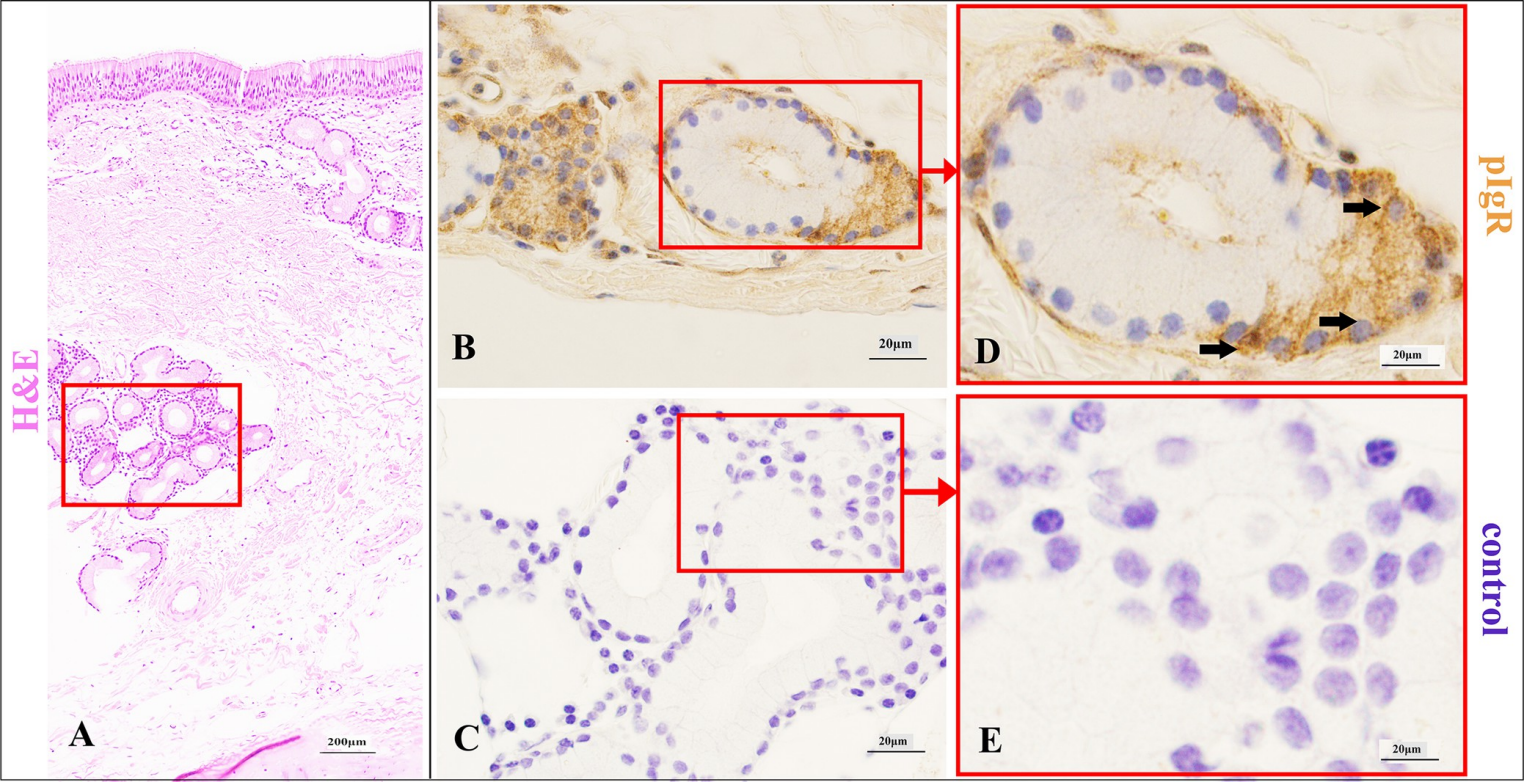
pIgR expression characteristics in the main bronchial glands of Bactrian camels.

**Fig 4:**
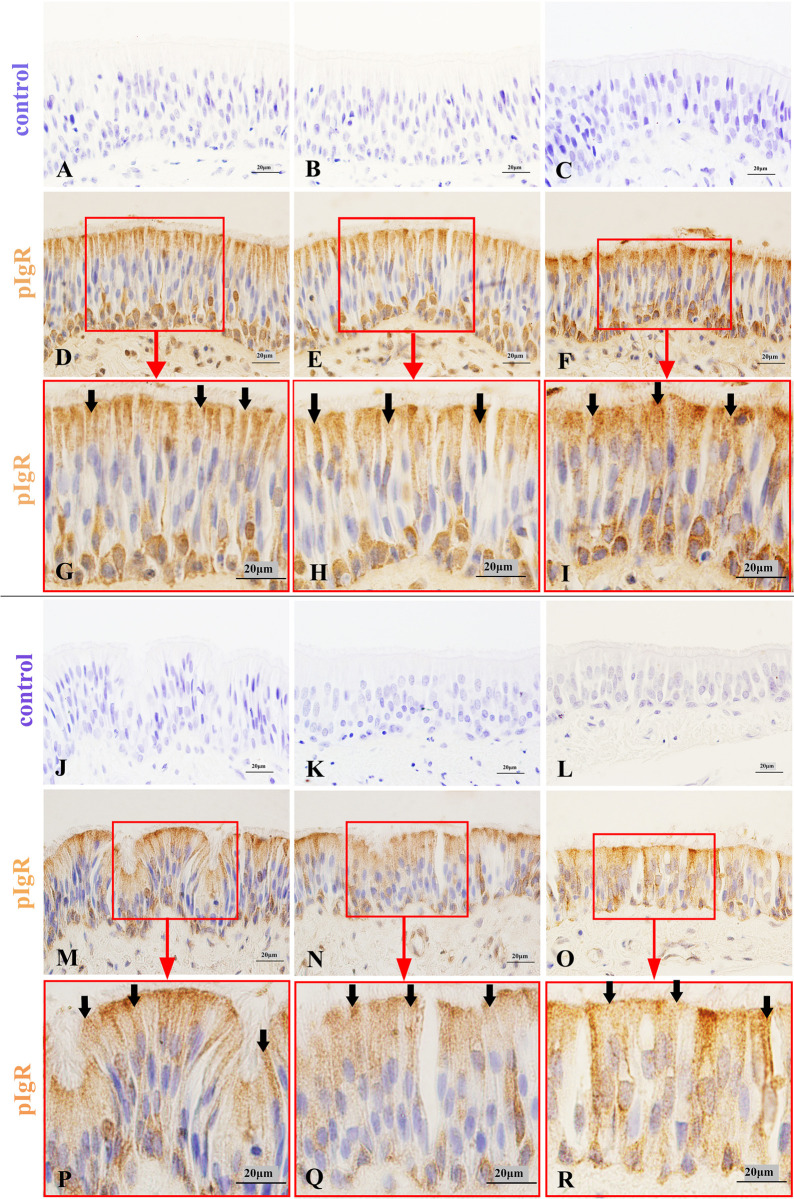
pIgR expression characteristics in the ciliated epithelial cells of different graded bronchi in Bactrian camels.

pIgR could be expressed in each bronchial branch of the lung conducting portion in Bactrian camels, including the dorsal, ventral, medial and lateral bronchiole systems. In the larger airways, the trachea and main bronchi, pIgR was highly expressed in the cytoplasm and membrane of ciliated cells, basal cells (Fig 2) and serous cells of bronchial glands (Fig 3). However, pIgR was not found in the goblet cells, endocrine cells and mucous cells of bronchial glands. In addition, as the bronchial glands gradually decreased, pIgR was mainly expressed in ciliated cells (Fig 4).

Histological characteristics of the main bronchi (A) and the main bronchial epithelium (red frame) in Bactrian camels, stained with haematoxylin and eosin (H&E). Original magnification: 100×; pIgR was highly expressed in the main bronchial ciliated epithelial cells (arrows), and also could be expressed in the basal cells (triangle arrows) (B), stained with pIgR-immunohistochemistry (IHC). Original magnification: 1000×; negative control of the main bronchial epithelium (C), stained with IHC. Original magnification: 1000×; pictures (D-E) were representative views of the main bronchial epithelium from pictures (B-C), and the original magnifications were all 1000×.

Histological characteristics of the main bronchi (A) and the main bronchial glands (red frame) in Bactrian camels, stained with haematoxylin and eosin (H&E). Original magnification: 100×; pIgR was highly expressed in the serous cells of the main bronchial glands (arrows) (B), stained with pIgR-immunohistochemistry (IHC). Original magnification: 1000×; the negative control of the main bronchial glands (C), stained with IHC. Original magnification: 1000×; pictures (D-E) were representative views of the main bronchial glands from pictures (B-C), and the original magnifications were all 1000×.

Pictures (A-C) were the negative control of trachea, main bronchi and lobar bronchi, stained with immunohistochemistry (IHC), and the original magnifications were all 1000×; pictures (D-F) were the pIgR expression characteristics in the ciliated epithelial cells (arrows) of trachea, main bronchi and lobar bronchi, stained with pIgR-IHC, and the original magnifications were all 1000×; pictures (G-I) were representative views of pictures (D-F), stained with pIgR-IHC, and the original magnifications were all 1000×; Pictures (J-L) were the negative control of segmental bronchi, sub-segmental bronchi and small bronchi, stained with immunohistochemistry (IHC), and the original magnifications were all 1000×; pictures (M-O) were the pIgR expression characteristics in the ciliated epithelial cells (arrows) of segmental bronchi, sub-segmental bronchi and small bronchi, stained with pIgR-IHC, and the original magnifications were all 1000×; pictures (P-R) were representative views of pictures (M-O), stained with pIgR-IHC, and the original magnifications were all 1000×.

### pIgR expression characteristics in the respiratory portion of Bactrian camel lungs

**Fig 5:**
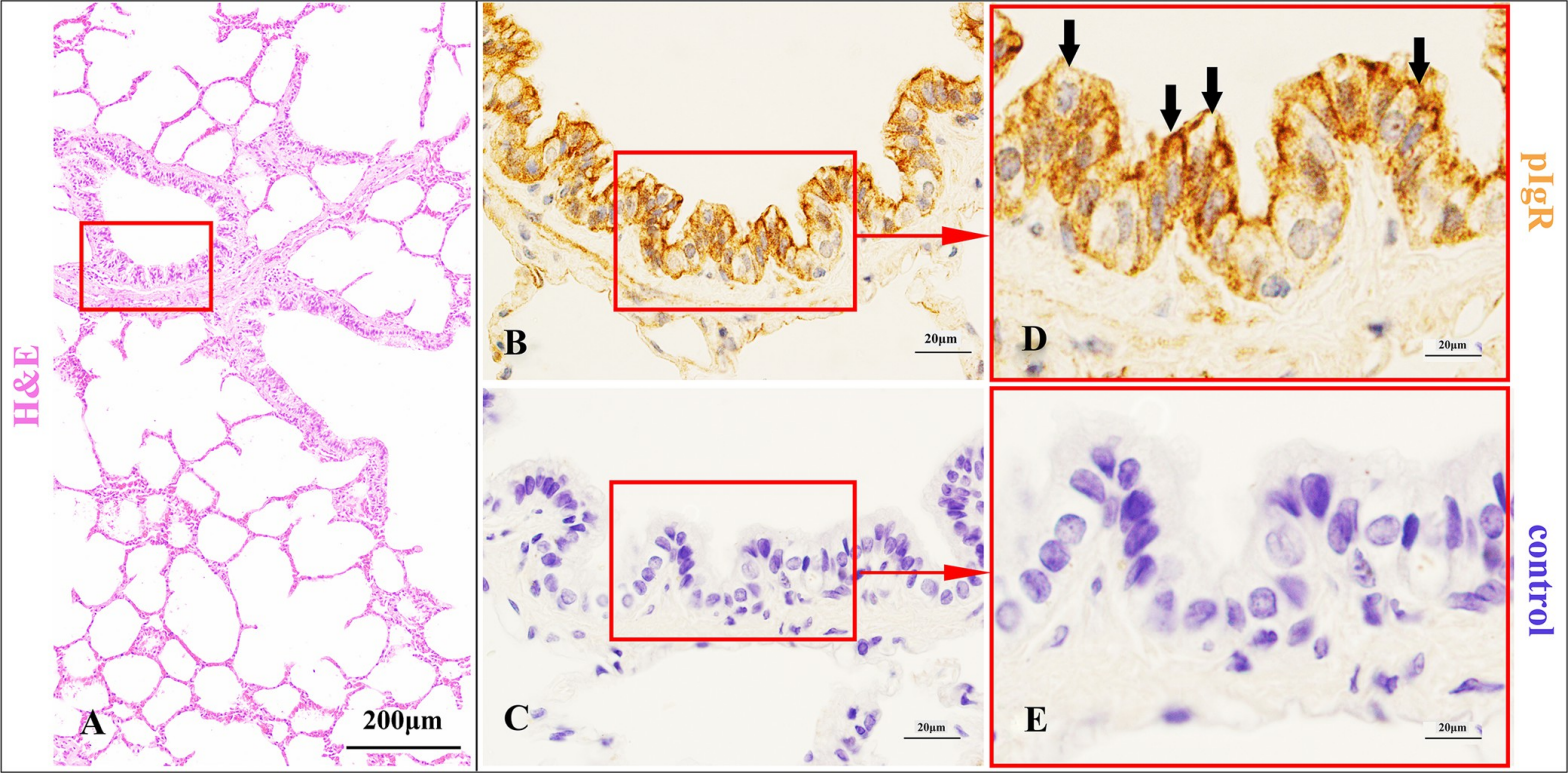
pIgR expression characteristics in terminal bronchial epithelium of Bactrian camels.

**Fig 6:**
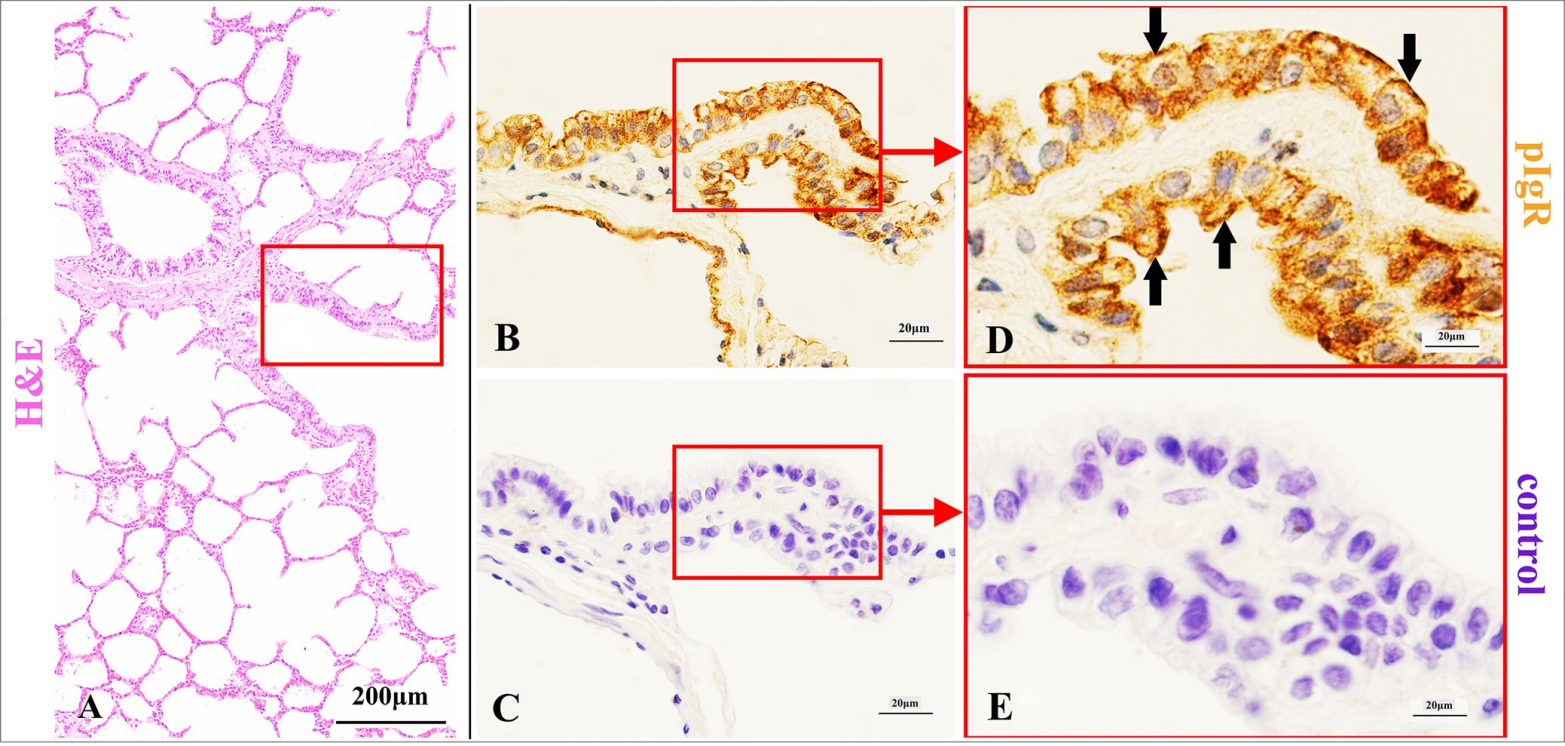
pIgR expression characteristics of respiratory bronchial epithelium in Bactrian camels.

**Fig 7:**
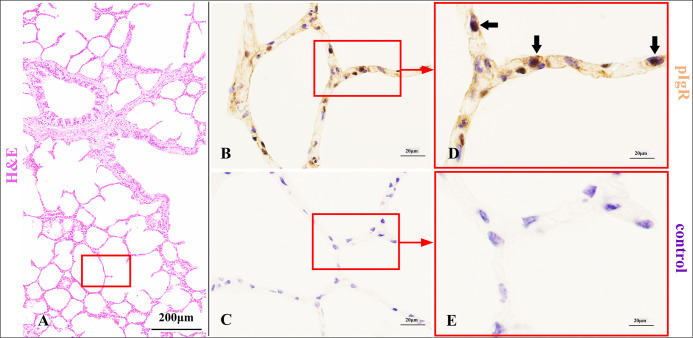
pIgR expression characteristics of pulmonary alveoli epithelium in Bactrian camels.

In the terminal bronchi and respiratory bronchi, pIgR was still primarily expressed in the cytoplasm and membrane of ciliated cells, and was also expressed in the cytoplasm and membrane of club cells (Figs 5 and 6). Moreover, in the pulmonary alveoli, pIgR was highly expressed in the cytoplasm and membrane of alveolar type 2 (AT2) cells (Fig 7), but absent in alveolar type 1 (AT1) cells.

Histological characteristics of lung respiratory portion (A) and terminal bronchi (red frame) in Bactrian camels, stained with haematoxylin and eosin (H&E). Original magnification: 100×; pIgR was mainly in the club cells (arrows) of terminal bronchi (B), stained with pIgR-immunohistochemistry (IHC). Original magnification: 1000×; negative control of terminal bronchi epithelium (C), stained with IHC. Original magnification: 1000×; pictures (D-E) were representative views of terminal bronchi epithelium from pictures (B-C), and the original magnifications were all 1000×.

Histological characteristics of lung respiratory portion (A) and respiratory bronchi (red frame) in Bactrian camels, stained with haematoxylin and eosin (H&E). Original magnification: 100×; pIgR was mainly expressed in the club cells (arrows) of respiratory bronchi (B), stained with pIgR-immunohistochemistry (IHC). Original magnification: 1000×; negative control of respiratory bronchi epithelium (C), stained with IHC. Original magnification: 1000×; pictures (D-E) were representative views of respiratory bronchi epithelium from pictures (B-C), and the original magnifications were all 1000×.

Histological characteristics of lung respiratory portion (A) and pulmonary alveoli (red frame) in Bactrian camels, stained with haematoxylin and eosin (H&E). Original magnification: 100×; pIgR expressed in the alveolar type 2 (AT2) cells (arrows) (B), stained with pIgR- immunohistochemistry (IHC). Original magnification: 1000×; negative control of AT2 cells (C), stained with IHC. Original magnification: 1000×; pictures (D-E) were representative views of AT2 cells from pictures (B-C), and the original magnifications were all 1000×.

### pIgR expression regularity in the ciliated cells of different bronchial branches in Bactrian camel lungs

**Fig 8:**
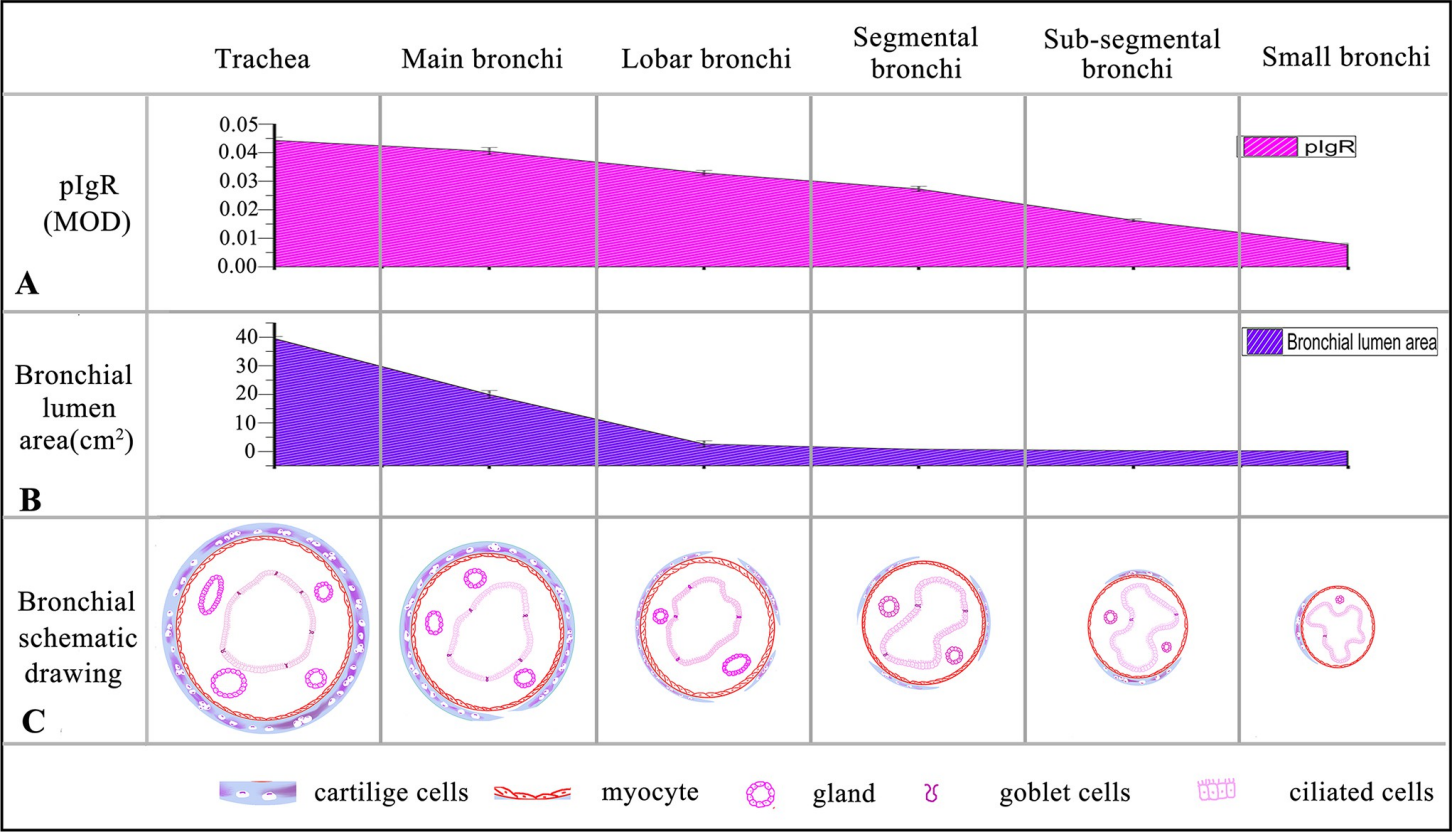
pIgR expression variation regularity in the ciliated epithelial cells of different graded bronchi in Bactrian camels.

The statistical results of pIgR (MOD) showed that as the number of bronchial branches increased, the pIgR expression level in bronchial ciliated cells was significantly decreased (p<0.05). For example, trachea > main bronchi > lobar bronchi > segmental bronchi > sub-segmental bronchi > small bronchi (Fig 8). In addition, in the different segmental bronchi at the same grade that diverged from the same lobar bronchi, pIgR expression level in ciliated cells of the larger luminal areas was significantly higher than that in the smaller areas (S2 File). For instance, in the right lungs, Acr > Aca in the cranial lobe; Acd > Acv in the accessory lobe; and D2 > D1 > D4 > D5 > D6, L2 > L1 > L3, M3 > M4 > M5 > M6, and V1 > V2 > V3 > V4 > V5 in the caudal lobe. In the left lungs, Acr < Aca in the cranial lobe; and D1 > D2 > D3 > D4 > D5 > D6, L2 > L1 > L3, M3 > M4 > M5 > M6, and V1 > V2 > V3 > V4 > V5 in the caudal lobe.

From trachea to small bronchi, pIgR expression level in the ciliated epithelial cells significantly declined (p<0.05) (A), and the corresponding bronchial luminal areas significantly decreased (p<0.05) (B), that is, trachea > main bronchi > lobar bronchi > segmental bronchi > sub-segmental bronchi > small bronchi; the schematic drawing of cross-sections of trachea, main bronchi, lobar bronchi, segmental bronchi, sub-segmental bronchi and small bronchi (C).

## Discussion

The results showed that in Bactrian camel, the lung mucosal epithelium was mainly consisted of ciliated cells, goblet cells, basal cells, endocrine cells, club cells, alveolar type 1 (AT1) cells and alveolar type 2 (AT2) cells, which was similar to in human [34] and other animals [35] including Dromedary camel [36], yak [37] and cattle [38], except in buffalo (without goblet cells) [37]. But unfortunately, pIgR as the critical structural part of the SIgA formation, there is still very little research on the mammalian lungs, only in human [4, 22–25], pig [26], rat [27, 28], mouse [29], and rhesus monkey [30]. While interestingly, in this study, pIgR was mainly highly expressed in ciliated cells (from trachea to respiratory bronchi), but not in goblet cells and endocrine cells of Bactrian camel lungs. These expression characteristics in the bronchial mucosal epithelium of Bactrian camels were similar to those in rats [27, 28], mice [29] and humans [4, 22–25]. In fact, ciliated cells are the most abundant cell type (approximately 50%-90%) [39] that constitutes the bronchial mucosal epithelium, and their half-life is up to 18 months [40], and more importantly, ciliated cells are the primary targets for some respiratory pathogens, including bacteria, viruses, and fungi, such as *Streptococcus pneumoniae*, *Pseudomonas aeruginosa*, SARS-CoV, Rhinovirus C and *Aspergillus flavus* [41–43]. Therefore, ciliated cells, as the major expression sites of pIgR, were the most critical for the major effector molecule of mucosal immune–SIgA to achieve transmembrane transport in the lung. Besides, in some reports, basal cells have been found to be negative for pIgR staining [4], but they were positive in our study. These results might be closely related to the transport direction of SIgA, because SIgA is transported by pIgR from the mucosal epithelial basal side into the luminal mucosal secretions, so basal cells, as the major constituent located at the epithelial basement, could be more convenient for SIgA transmembrane transport.

The statistical results showed that from the trachea to small bronchi of Bactrian camels, the pIgR expression level in the bronchial ciliated cells significantly declined (p<0.05), and the bronchial luminal areas obviously decreased (p<0.05). Similarly, in the different segmental bronchi that simultaneously diverged from the same lobar bronchi, the pIgR expression level in the ciliated cells of the larger luminal areas was distinctly higher than that in the smaller areas (p<0.05). This inhomogeneous characteristics of pIgR expression in the bronchial ciliated cells of Bactrian camels were similar to the respiratory tract in humans [21] and mice [44]. Simultaneously, in Bactrian camels, as the number of bronchial branches increased, the bronchial epithelium changed from pseudostratified to simple epithelium (columnar or cubic), and the number of ciliated cells gradually decreased, were identical with yak [37] and least shrew [45], on the contrary, the airflow cleanliness of the corresponding bronchial cross-sections increased because the bronchial luminal surface gradually cleaned by the nonspecific physical adhesion and natural immune clearance functions of bronchial epithelial cells [39, 46–48]. Therefore, the regular variations among the pIgR expression levels of the different bronchial ciliated cells, the different bronchial luminal areas, and the numbers of different bronchial ciliated cells in Bactrian camels, were all consistent.

Moreover, pIgR was also found in the serous cells of bronchial glands, bronchial club cells, and AT2 cells in Bactrian camel lungs. These findings are similar to the reports that pIgR is highly expressed in the serous cells of bronchial glands in rats [27, 28], mice [29], pigs [26] and humans [4, 22–25], and is also expressed in the non-ciliated cells of the bronchiole epithelium and some AT2 cells of humans [4, 49]. Unexpectedly, although pIgR can be expressed in human AT1 cells [49] and mucous cells of the bronchial glands [24], no positive expression was observed in two of them in this study. According to some reports, the pIgR expression characteristics in individual cells can be affected by certain cytokines, hormones, dietary factors and ages [50, 51], but pIgR expression is closely related to the microbial stimulation, especially in the mucosal epithelium [18]. For example, monocolonization of germ-free mice with the commensal bacterium *Bacteroides thetaiotaomicron* resulted in increased pIgR expression in intestinal epithelial cells [52], pIgR expression in the intestinal epithelium can be regulated by the symbiotic bacteria of this intestinal segment [53], and pIgR expression in the colon epithelium can be upregulated by the microbial product butyrate [54]. Besides, pIgR expression can also be regulated by the signaling pathways, including TLR4 [55, 56], Notch [8], JAK-STAT [18], NF-κB [57] and other factors. This study provided a basis for further exploring the patterns of pIgR expression in the respiratory tract of Bactrian camels.

Above all, pIgR expression characteristics in Bactrian camel lungs clearly reflected the fact that pIgR is selectively expressed in mucosal and glandular epithelial cells [58, 59]. Importantly, in Bactrian camel lungs, pIgR could be simultaneously expressed in ciliated cells, basal cells, club cells, AT2 cells and serous cells of bronchial glands. These types of cells, could guarantee the continuous and efficient transport of immunoglobulins (i.e., pIgA) across the membrane, resulting in the timely formation of the immune defense barrier.

## Conclusions

In this study, the characteristics of pIgR expression in the conducting and respiratory portions of Bactrian camel lungs were observed and analysed through immunohistochemistry, micro-image analysis and statistical methods. The results demonstrated that the bronchial ciliated cells continuously distributed throughout the whole bronchial tree mucosa were the major expression sites of pIgR, but pIgR could also be expressed in basal cells, serous cells of bronchial glands, club cells and alveolar type 2 cells, which would facilitate secretory immunoglobulin A (SIgA) transmembrane transport by pIgR and form an intact protective barrier. Moreover, the pIgR expression level in ciliated cells was positively correlated with the bronchial luminal areas, but negatively correlated with the airflow cleanliness through the bronchial cross-sections, which signified that the pIgR expression level in the bronchial epithelium was inhomogeneous. Our study provided a foundation for further exploring the regulatory behaviour of immunoglobulins (i.e., SIgA) after transport across the membrane by pIgR in Bactrian camel lungs.

## Supporting information

S1 File The bronchial luminal areas of each bronchial branch in Bactrian camels. As the bronchial branches extended, the relative luminal areas significantly declined (p<0.05); that is, trachea > main bronchi > lobar bronchi > segmental bronchi > sub-segmental bronchi > small bronchi. And in the different branches at the same grade from the same lobar bronchi, the areas of the larger lumens were significantly higher than those of the smaller lumens.
(DOCX)

S2 File The MOD results of pIgR expression level in the ciliated cells of each bronchial branch in Bactrian camels. As the number of bronchial branches increased, the MOD result of pIgR expression level in bronchial ciliated cells was significantly decreased (p<0.05). that is, trachea > main bronchi > lobar bronchi > segmental bronchi > sub-segmental bronchi > small bronchi. And in the different segmental bronchi at the same grade that diverged from the same lobar bronchi, the MOD result of pIgR expression level in ciliated cells of the larger luminal areas was significantly higher than that in the smaller areas.
(DOCX)

S3 File (PDF)

## References

[1] Eiffert H, Quentin E, Decker J, Hillemeir S, Hufschmidt M, Klingmüller D, et al. The primary structure of human free secretory component and the arrangement of disulfide bonds. Hoppe-Seyler’s Zeitschrift für physiologische Chemie. 1984;365:1489. 6526384

[2] Mostov KE, Friedlander M, Blobel G. The receptor for transepithelial transport of IgA and IgM contains multiple immunoglobulin-like domains. Nature. 1984;308:37–43. doi: 10.1038/308037a0 6322002

[3] Bruno MEC, Kaetzel CS. Long-Term Exposure of the HT-29 Human intestinal epithelial cell line to TNF causes sustained up-regulation of the polymeric Ig receptor and proinflammatory genes through transcriptional and posttranscriptional mechanisms. The Journal of Immunology. 2005;174:7278–7284. doi: 10.4049/jimmunol.174.11.7278 15905574

[4] Rossel M, Brambilla E, Billaud M, Vuitton DA, Blanc-Jouvan F, Biichle S, et al. Nonspecific increased serum levels of secretory component in lung tumors: relationship to the gene expression of the transmembrane receptor form. American Journal of Respiratory Cell and Molecular Biology. 1993;9:341–346. doi: 10.1165/ajrcmb/9.3.341 8398172

[5] Martín MG, Wang JF, Li TWH, Lam JT, Gutierrez EM, R SSV, et al. Characterization of the 5’-flanking region of the murine polymeric IgA receptor gene. American Journal of Physiology. 1998;275:G778. doi: 10.1152/ajpgi.1998.275.4.G778 9756509

[6] Jenkins SL, Wang J, Vazir M, Vela J, Sahagun O, Gabbay P, et al. Role of passive and adaptive immunity in influencing enterocyte-specific gene expression. American Journal of Physiology. Gastrointestinal and liver Physiology. 2003;285(4):G714–25. doi: 10.1152/ajpgi.00130.2003 12969828

[7] Huling S, Fournier GR, Feren A, Chountharapai A, Jones AL. Ontogeny of the secretory immune system: maturation of a functional polymeric immunoglobulin receptor regulated by gene expression. Proceeding of the National Academy of Sciences of the United States of America. 1992;89(10):4260–4264. doi: 10.1073/pnas.89.10.4260 PMC49061 1374892

[8] Ocak S, Pedchenko TV, Chen H, Harris FT, Qian J, Polosukhin V, et al. Loss of polymeric immunoglobulin receptor expression is associated with lung tumourigenesis. European Respiratory Journal. 2012;39:1171–1180. doi: 10.1183/09031936.00184410 21965228 PMC3717253

[9] Isaacson P. Immunoperoxidase study of the secretory immuno-globulin system in colonic neoplasia. Journal of Clinical Pathology. 1982;35:14–25. doi: 10.1136/jcp.35.1.14 6801094 PMC497442

[10] Robinson JK, Blanchard TG, Levine AD, Emancipator SN, Lamm ME. A mucosal IgA-mediated excretory immune system in vivo. Journal of Immunology. 2001; 166:3688–3692. doi: 10.4049/jimmunol.166.6.3688 11238608

[11] Wijburg OLC, Uren TK, Simpfendorfer K, Johansen FE, Brandtzaeg P, Strugnell RA. Innate secretory antibodies protect against natural Salmonella typhimurium infection. Journal of Experimental Medicine. 2006;203:21–26. doi: 10.1084/jem.20052093 16390940 PMC2118088

[12] Huang YT, Wright A, Gao X, Kulick L, Yan H, Lamm ME. Intraepithelial cell neutralization of HIV-1 replication by IgA. Journal of Immunology. 2005;174:4828–4835. doi: 10.4049/jimmunol.174.8.4828 15814709

[13] Kaetzel CS, Robinson JK, Chintalacharuvu KR, Vaerman JP, Lamm ME. The polymeric immunoglobulin receptor (secretory component) mediates transport of immune complexes across epithelial cells: a local defense function for IgA. Proceedings of the National Academy of Sciences of United States of America. 1991;88:8796–8800. doi: 10.1073/pnas.88.19.8796 1924341 PMC52597

[14] Underdown BJ, Switzer I, Jackson GD. Rat secretory component binds poorly to rodent IgM. Journal of Immunology. 1992;149:487–491. 1624795

[15] Mestecky J, Kulhavy R, Wright GP, Tomana M. Studies on human secretory immunoglobulin A. VI. Cyanogen bromide cleavage. Journal of Immunology. 1974; 113:404–412. 4208924

[16] Wieland WH, Orzáez D, Lammers A, Parmentier HK, Verstegen MW, Schots A. A functional polymeric immunoglobulin receptor in chicken (Gallus gallus) indicates ancient role of secretory IgA in mucosal immunity. Biochemical Journal. 2004;380:669–676. doi: 10.1042/BJ20040200 14992684 PMC1224204

[17] Brandtzaeg P, Carlsen HS, Halstensen TS. The B-cell system in inflammatory bowel disease. Advances in experimental medicine and biology. 2006; 579:149–167. doi: 10.1007/0-387-33778-4_10 16620017

[18] Kaetzel CS. The polymeric immunoglobulin receptor: bridging innate and adaptive immune responses at mucosal surfaces. Immunological Reviews. 2010;206:83–99. doi: 10.1111/j.0105-2896.2005.00278.x 16048543

[19] de Oliveira IR, de Araújo AN, Bao SN, Giugliano LG. Binding of lactoferrin and free secretory component to enterotoxigenic Escherichia coli. FEMS microbiology letters. 2001;203:29–33. doi: 10.1111/j.1574-6968.2001.tb10816.x 11557136

[20] Phalipon A, Cardona A, Kraehenbuhl JP, Edelman L, Sansonetti PJ, Corthesy B. Secretory component: a new role in secretory IgA-mediated immune exclusion in vivo. Immunity. 2002;17:107–115. doi: 10.1016/s1074-7613(02)00341-2 12150896

[21] Zhang JR, Mostov KE, Lamm ME, Nanno M, Shimida SI, Ohwaki M, et al. The polymeric immunoglobulin receptor translocates pneumococci across human nasopharyngeal epithelial cells. Cell. 2000;102:827–837. doi: 10.1016/s0092-8674(00)00071-4 11030626

[22] Du RH, Richmond BW, Blackwell Jr TS, Cates JM, Massion PP, Ware LB, et al. Secretory IgA from submucosal glands does not compensate for its airway surface deficiency in chronic obstructive pulmonary disease. Virchows Arch. 2015;467:657–665. doi: 10.1007/s00428-015-1854-0 26432569 PMC5081073

[23] Ferkol T, Kaetzel CS, Davis PB. Gene transfer into respiratory epithelial cells by targeting the polymeric immunoglobulin receptor. Journal of Clinical Investigation. 1993;92:2394–2400. doi: 10.1172/JCI116845 8227356 PMC288422

[24] Pilette C, Godding V, Kiss R, Delos M, Verbeken E, Decaestecker C, et al. Reduced epithelial expression of secretory component in small airways correlates with airflow obstruction in chronic obstructive pulmonary disease. American Journal of Respiratory and Critical Care Medicine. 2001;163:185. doi: 10.1164/ajrccm.163.1.9912137 11208645

[25] Pilette C, Ouadrhiri Y, Godding V, Vaerman JP, Sibille Y. Lung mucosal immunity: immunoglobulin-A revisited. European Respiratory Journal. 2001;19:571–588. doi: 10.1183/09031936.01.00228801 11589357

[26] Guzman-Bautista ER, Ramirez-Estudillo MC, Rojas-Gomez OI. Vega-Lopez MA. Tracheal and bronchial polymeric immunoglobulin secretory immune system (PISIS) development in a porcine model. Developmental and Comparative Immunology. 2015; 53:271–282. doi: 10.1016/j.dci.2015.07.010 26188097

[27] Ferkol T, Perales JC, Eckman E, Kaetzel CS, Hanson RW, Davis PB. Gene transfer into the airway epithelium of animals by targeting the polymeric immunoglobulin receptor. Journal of Clinical Investigation. 1995;95:493–502. doi: 10.1172/JCI117690 7860731 PMC295497

[28] Liu DY, Jiang T, Wang S, Cao X. Effect of hyperoxia on pulmonary SIgA and its components, IgA and SC. Journal of Clinical Immunology. 2013;33:1009–1017. doi: 10.1007/s10875-013-9891-4 23579770 PMC3682104

[29] Jaffar Z, Ferrini ME, Herritt LA, Roberts K. Cutting edge: lung mucosal Th17-mediated responses induce polymeric Ig receptor expression by the airway epithelium and elevate secretory IgA levels. Journal of Immunology. 2009;182:4507–4511. doi: 10.4049/jimmunol.0900237 19342622 PMC2740792

[30] Li D, Wang FJ, Yu L, Yao WR, Cui YF, Yang GB. Expression of pIgR in the tracheal mucosa of SHIV/SIV-infected rhesus macaques. Zoological Research. 2017; 38(1):44–48. doi: 10.13918/j.issn.2095-8137.2017.007 28271669 PMC5368380

[31] Zhao XX. Ecophysiology and Reproduction of the Camelidae. Gansu Science and Technology Press, China. 1995.

[32] He WH, Zhang WD, Cheng CC, Li JF, Wu XP, Li M, et al. The distributive and structural characteristics of bronchus-associated lymphoid tissue (BALT) in Bactrian camels (Camelus bactrianus). PeerJ. 2019;7:e6571. doi: 10.7717/peerj.6571 30881767 PMC6417404

[33] Jia S. Gene cloning, bioinformatics analysis of Bactrian camel PIGR and FCGRT and study of their expression features in tonsils. Vol. Doctor. Gansu Agricultural University, Lanzhou, Gansu, China. 2017.

[34] Cheng LZ, Zhong CP, Cai WQ. Contemporary Histology. Shanghai scientific and technological literature publishing house, Shanghai, China, 2003.

[35] Chen YX, Cui Y. Animal Anatomy histology and embryology (Full colour). China Agricultural Publishing House. Beijing, China, 2020.

[36] Ahmad Reza Raji. Histological study of lung parenchyma of the One-humped Camel(*Camelus dromedaries*). Journal of Applied Animal Research. 2006;30(1):37–40.

[37] Yang B, Yu SJ, Cui Y, He JF, Jin XH, Wang. Morphological analysis of the lung of neonatal yak. Anatomia Histologia Embryologia. 2010;39:138–151. doi: 10.1111/j.1439-0264.2009.00988.x 20070291

[38] Lovannitti B, Pirie HM, Wright N. Scanning electron microscopic study of the lower respiratory tract in calves and adult cattle. Research in Veterinary Science. 1985 Jan;38(1):80–7. 3975486

[39] Ganesan S, Comstock AT, Sajjan US. Barrier function of airway tract epithelium. Tissue Barriers. 2013;1:e24997. doi: 10.4161/tisb.24997 24665407 PMC3783221

[40] Rawlins EL, Hogan BL. Ciliated epithelial cell lifespan in the mouse trachea and lung. American Journal of Physiology-Lung Cellular and Molecular Physiology. 2008;295:L231–234. doi: 10.1152/ajplung.90209.2008 18487354 PMC2494792

[41] Kuek LE, Lee RJ. First contact: the role of respiratory cilia in host-pathogen interactions in the airways. Am J Physiol Lung Cell Mol Physiol. 2020;319(4): L603–L619. doi: 10.1152/ajplung.00283.2020 32783615 PMC7516383

[42] Ahn JH, Kim JM, Hong SP, Choi SY, Yang MJ, Ju YS, et al. Nasal ciliated cells are primary targets for SARS-CoV-2 replication in the early stage of COVID-19. J Clin Invest. 2021;131(13):e148517. doi: 10.1172/JCI148517 34003804 PMC8245175

[43] Griggs TF, Bochkov YA, Basnet S, Pasic T, Brockman-Schneider RA, Palmenberg AC, et al. Rhinovirus C targets ciliated airway epithelial cells. Respir Res. 2017;18(1):84. doi: 10.1186/s12931-017-0567-0 28472984 PMC5418766

[44] Pausder A, Fricke J, Schughart K, Schreiber J, Strowing T, Bruder D, et al. Exogenous and endogenous triggers differentially stimulate pIgR expression and antibacterial secretory immunity in the murine respiratory tract. Lung. 2021;26. doi: 10.1007/s00408-021-00498-8 34825965 PMC8881272

[45] Arodaki F, Khamas W, Darmani N, Tikriti M. Histological characteristics of the tracheobronchial tree of the least shrew (cryptotis parva). Anat Histol Embryol. 2017;46(4):405–409. doi: 10.1111/ahe.12272 28466485

[46] Rokicki W, Rokicki M, Wojtacha J, Dzeljijli A. The role and importance of club cells (Clara cells) in the pathogenesis of some respiratory diseases. Kardiochir Torakochirurgia Pol. 2016;13:26–30. doi: 10.5114/kitp.2016.58961 27212975 PMC4860431

[47] Weitnauer M, Mijosek V, Dalpke AH. Control of local immunity by airway epithelial cells. Mucosal Immunology. 2016;9:287–298. doi: 10.1038/mi.2015.126 26627458

[48] Widdicombe JH, Wine JJ. Airway Gland Structure and Function. Physiological Reviews. 2015;95:1241–1319. doi: 10.1152/physrev.00039.2014 26336032

[49] Yang X. Study on the binding sites of human Fcα/μR and its ligand. Vol. Doctor. Peking Union Medical College, Beijing, China. 2012.

[50] Trevisi P, Gandolfi G, Priori D, Messori S, Colombo M, Mazzoni M, et al. Age-related expression of the polymeric immunoglobulin receptor (pIgR) in the gastric mucosa of young pigs. PLOS One. 2013;8(11):e81473. doi: 10.1371/journal.pone.0081473 24236214 PMC3827463

[51] Hinojosa E, Boyd AB, Orihuela CJ. Age-associated inflammation and toll-like receptor dysfunction prime the lungs for pneumococcal pneumonia. J Infect Dis. 2009;200(4):546–54. doi: 10.1086/600870 19586419 PMC3102250

[52] Johansen FE, Kaetzel CS. Regulation of the polymeric immunoglobulin receptor and IgA transport: new advances in environmental factors that stimulate pIgR expression and its role in mucosal immunity. Mucosal Immunology. 2011,4:598–602. doi: 10.1038/mi.2011.37 21956244 PMC3196803

[53] Hooper LV, Wong WH, Thelin A, Hansson L, Falk PG, Gordon JI. Molecular analysis of commensal host-microbial relationships in the intestine. Science. 2001;291:881–884. doi: 10.1126/science.291.5505.881 11157169

[54] Kvale D, Brandtzaeg P. Constitutive and cytokine induced expression of HLA molecules, secretory component, and intercellular adhesion molecule-1 is modulated by butyrate in the colonic epithelial cell line HT-29. Gut. 1995;36:737–742. doi: 10.1136/gut.36.5.737 7797124 PMC1382679

[55] Blanch VJ, Piskurich JF, Kaetzel CS. Cutting edge: coordinate regulation of IFN regulatory factor-1 and the polymeric Ig receptor by proinflammatory cytokines. Journal of Immunology. 1999;162:1232–1235. 9973374

[56] Kaetzel CS. Polymeric Ig receptor: defender of the fort or Trojan horse? Current Biology. 2001;11:R35–R38. doi: 10.1016/s0960-9822(00)00041-5 11166195

[57] Schjerven H, Brandtzaeg P, Johansen FE. A novel NF-kB/Rel site in intron 1 cooperates with proximal promoter elements to mediate TNF α-induced transcription of the human polymeric Ig receptor. Journal of Immunology. 2001; 167: 6412–6420. doi: 10.4049/jimmunol.167.11.6412 11714807

[58] Hempen PM, Phillips KM, Conway PS, Sandoval KH, Schneeman TA, Wu HJ, et al. Transcriptional Regulation of the Human Polymeric Ig Receptor Gene: Analysis of Basal Promoter Elements. The Journal of Immunology. 2002;169:1912–1921. doi: 10.4049/jimmunol.169.4.1912 12165516

[59] Schneeman TA, Bruno MEC, Schjerven H, Johansen FE, Chady L, Kaetzel CS. Regulation of the Polymeric Ig Receptor by Signaling through TLRs 3 and 4: Linking Innate and Adaptive Immune Responses. The Journal of Immunology. 2005;175:376–384. doi: 10.4049/jimmunol.175.1.376 15972671

