# Supplementary material for: Expression characteristics of polymeric immunoglobulin receptor in Bactrian camel (Camelus bactrianus) lungs
Source: PLoS One. 2022 Mar 4;17(3):e0264815. doi: 10.1371/journal.pone.0264815 (PMC8896721; doi:10.1371/journal.pone.0264815)
Supplement: S1 File — As the bronchial branches extended, the relative luminal areas significantly declined (p<0.05); that is, trachea > main bronchi > lobar bronchi > segmental bronchi > sub-segmental bronchi > small bronchi. And in the different branches at the same grade from the same lobar bronchi, the areas of the larger lumens were significantly higher than those of the smaller lumens. (DOCX) [file pone.0264815.s001.docx]

**S1 File. The bronchial luminal areas of each bronchial branch in Bactrian camels.**

| Bronchial tree of Bactrian camels | | | The bronchial luminal areas (cm^2^) |
| --- | --- | --- | --- |
| Trachea | | | 39.5271±0.6902a |
| Right main bronchi | | | 19.9397±1.3910b |
| Left main bronchi | | | 19.9402±0.9786b |
| Right cranial lobe | | Lobar bronchi | 2.6265±0.1524a |
|  |  | Cranial segmental bronchi (Acr) | 0.8133±0.5120b |
|  |  | Caudal segmental bronchi (Aca) | 0.3449±0.2544c |
| Accessory lobe | | Lobar bronchi | 0.2998±0.0193a |
|  |  | Ventral segmental bronchi (Acv) | 0.2548±0.0161b |
|  |  | Dorsal segmental bronchi(Acd) | 0.2052±0.0180c |
| Right caudal lobe | Dorsal segmental bronchi (D) | D1 | 1.8350±0.3405a |
|  |  | D2 | 1.0148±0.1025b |
|  |  | D4 | 0.6219±0.0439c |
|  |  | D5 | 0.2872±0.0504d |
|  |  | D6 | 0.1497±0.0232e |
|  | Ventral segmental bronchi (V) | V1 | 0.3398±0.0581a |
|  |  | V2 | 0.3138±0.0612a |
|  |  | V3 | 0.2752±0.0547b |
|  |  | V4 | 0.2341±0.0474c |
|  |  | V5 | 0.1688±0.0432d |
|  | Lateral segmental bronchi (L) | L1 | 2.4023±0.3135a |
|  |  | L2 | 1.8445±0.2215b |
|  |  | L3 | 1.3364±0.1548c |
|  | Medial segmental bronchi (M) | M3 | 0.1233±0.0106a |
|  |  | M4 | 0.1121±0.0142b |
|  |  | M5 | 0.0943±0.0151c |
|  |  | M6 | 0.0725±0.0159d |

| Left cranial lobe | | Lobar bronchi | 2.9366±0.2459a |
| --- | --- | --- | --- |
|  |  | Cranial segmental bronchi (Acr) | 0.4102±0.0490c |
|  |  | Caudal segmental bronchi (Aca) | 0.9468±0.1008b |
| Left caudal lobe | Dorsal segmental bronchi (D) | D1 | 1.8928±0.2672a |
|  |  | D2 | 0.9632±0.1027b |
|  |  | D3 | 0.7974±0.0727c |
|  |  | D4 | 0.5756±0.0602d |
|  |  | D5 | 0.2738±0.0347e |
|  |  | D6 | 0.1475±0.0225f |
|  | Ventral segmental bronchi (V) | V1 | 0.3161±0.0460a |
|  |  | V2 | 0.2778±0.0550b |
|  |  | V3 | 0.2354±0.0419c |
|  |  | V4 | 0.1841±0.0248d |
|  |  | V5 | 0.1387±0.0247e |
|  | Lateral segmental bronchi (L) | L1 | 2.3281±0.3106a |
|  |  | L2 | 1.7315±0.1924b |
|  |  | L3 | 1.2739±0.1343c |
|  | Medial segmental bronchi (M) | M3 | 0.1275±0.0108a |
|  |  | M4 | 0.1149±0.0121b |
|  |  | M5 | 0.0940±0.0147c |
|  |  | M6 | 0.0680±0.0146d |
