# Supplementary material for: Expression characteristics of polymeric immunoglobulin receptor in Bactrian camel (Camelus bactrianus) lungs
Source: PLoS One. 2022 Mar 4;17(3):e0264815. doi: 10.1371/journal.pone.0264815 (PMC8896721; doi:10.1371/journal.pone.0264815)
Supplement: S2 File — As the number of bronchial branches increased, the MOD result of pIgR expression level in bronchial ciliated cells was significantly decreased (p<0.05). that is, trachea > main bronchi > lobar bronchi > segmental bronchi > sub-segmental bronchi > small bronchi. And in the different segmental bronchi at the same grade that diverged from the same lobar bronchi, the MOD result of pIgR expression level in ciliated cells of the larger luminal areas was significantly higher than that in the smaller areas. (DOCX) [file pone.0264815.s002.docx]

**S2 File. The MOD results of pIgR expression level in the ciliated cells of each bronchial branch in Bactrian camels.**

| Bronchial tree of Bactrian camels | | | pIgR(MOD) |
| --- | --- | --- | --- |
| Trachea | | | 0.0443±0.0011a |
| Right main bronchi | | | 0.0405±0.0013b |
| Left main bronchi | | | 0.0406±0.0028b |
| Right cranial lobe | | Lobar bronchi | 0.0329±0.0009a |
|  |  | Cranial segmental bronchi (Acr) | 0.0314±0.0012b |
|  |  | Caudal segmental bronchi (Aca) | 0.0216±0.0017c |
| Accessory lobe | | Lobar bronchi | 0.0283±0.0012a |
|  |  | Ventral segmental bronchi (Acv) | 0.0180±0.0004b |
|  |  | Dorsal segmental bronchi(Acd) | 0.0140±0.0005c |
| Right caudal lobe | Dorsal segmental bronchi (D) | D1 | 0.0204±0.0010b |
|  |  | D2 | 0.0295±0.0019a |
|  |  | D4 | 0.0166±0.0010c |
|  |  | D5 | 0.0138±0.0002d |
|  |  | D6 | 0.0093±0.0007e |
|  | Ventral segmental bronchi (V) | V1 | 0.0204±0.0010a |
|  |  | V2 | 0.0180±0.0004b |
|  |  | V3 | 0.0111±0.0006c |
|  |  | V4 | 0.0078±0.0005d |
|  |  | V5 | 0.0048±0.0003e |
|  | Lateral segmental bronchi (L) | L1 | 0.0243±0.0020b |
|  |  | L2 | 0.0324±0.0012a |
|  |  | L3 | 0.0214±0.0016c |
|  | Medial segmental bronchi (M) | M3 | 0.0039±0.0001a |
|  |  | M4 | 0.0035±0.0001b |
|  |  | M5 | 0.0024±0.0001c |
|  |  | M6 | 0.0020±0.0001d |

| Left cranial lobe | | Lobar bronchi | 0.0330±0.0009a |
| --- | --- | --- | --- |
|  |  | Cranial segmental bronchi (Acr) | 0.0311±0.0009b |
|  |  | Caudal segmental bronchi (Aca) | 0.0223±0.0014c |
| Left caudal lobe | Dorsal segmental bronchi (D) | D1 | 0.0233±0.0009a |
|  |  | D2 | 0.0210±0.0009b |
|  |  | D3 | 0.0195±0.0008c |
|  |  | D4 | 0.0163±0.0007d |
|  |  | D5 | 0.0081±0.0030e |
|  |  | D6 | 0.0163±0.0055f |
|  | Ventral segmental bronchi (V) | V1 | 0.0204±0.0010a |
|  |  | V2 | 0.0180±0.0004b |
|  |  | V3 | 0.0113±0.0005c |
|  |  | V4 | 0.0080±0.0004d |
|  |  | V5 | 0.0049±0.0003e |
|  | Lateral segmental bronchi (L) | L1 | 0.0273±0.0018b |
|  |  | L2 | 0.0326±0.0011a |
|  |  | L3 | 0.0226±0.0014c |
|  | Medial segmental bronchi (M) | M3 | 0.0039±0.0001a |
|  |  | M4 | 0.0034±0.0001b |
|  |  | M5 | 0.0025±0.0001c |
|  |  | M6 | 0.0021±0.0001d |
