# Supplementary material for: Expression characteristics of polymeric immunoglobulin receptor in Bactrian camel (Camelus bactrianus) lungs
Source: PLoS One. 2022 Mar 4;17(3):e0264815. doi: 10.1371/journal.pone.0264815 (PMC8896721; doi:10.1371/journal.pone.0264815)
Supplement: S3 File — (PDF) [file pone.0264815.s003.pdf]

动物伦理审查同意书

Affidavit of Approval of Animal Ethical and Welfare

编号 Approval No.	GSAU-AEW-2016-0010

本《动物实验方案》经过实验动物伦理委员会审核，符合动物保护、动物福利和伦理原则，符合国家实验动物福利伦理的相关规定。方案的相关信息如下：

The animal use protocol listed below has been reviewed and approved by the Animal Ethical and Welfare Committee (AEWC).

课题名称 Protocol Title	双峰驼呼吸道黏膜免疫形态学及免疫相关因子的研究<br>The morphology and immune-related factors of mucosal immune in respiratory tract of Bactrian camels				
申请人 Applicant	何晚红<br>Wan-hong He	职称/学位 Title/Degree	博士<br>PhD.	邮箱 Email	hwh_456@163.com
课题负责人 Principle Investigator(PI)	王雯慧<br>Wen-Hui Wang	职称/学位 Title/Degree	教授<br>Prof.	邮箱 Email	wwh777@126.com
院系（部门） Department	动物医学院<br>College of Veterinary Medicine			申请日期 Application date	2016.12.31
动物种系 Species or Strains	阿拉善双峰驼<br>Bactrian Camels (Camelus bactrianus)			动物数量 Quantity	27
计划执行时间 Period of Protocol	2016.12.31-2019.12.31	实验动物编号 Number of Animal permit		No. SCXK (Gan)：No	
审查意见 Results of inspection	☑符合动物福利伦理要求，可以进行实验 Agree<br>☐调整方案后，可以进行实验 Agree after modify				

甘肃农业大学动物医学院实验动物伦理委员会

Animal Ethical and Welfare Committee of College Veterinary Medicine of GSAU

主席 (Chairman): [illegible]

日期 (Date): 21/3-2018
